# Stemness and chemoresistance in epithelial ovarian carcinoma cells under shear stress

**DOI:** 10.1038/srep26788

**Published:** 2016-06-01

**Authors:** Carman K. M. Ip, Shan-Shan Li, Matthew Y. H. Tang, Samuel K. H. Sy, Yong Ren, Ho Cheung Shum, Alice S. T. Wong

**Affiliations:** 1 School of Biological Sciences, University of Hong Kong, Pokfulam Road, Hong Kong; 2 Department of Mechanical Engineering, University of Hong Kong, Pokfulam Road, Hong Kong; 3 Department of Mechanical, Materials & Manufacturing Engineering, University of Nottingham Ningbo China, China

## Abstract

One of greatest challenges to the successful treatment of cancer is drug resistance. An exciting approach is the eradication of cancer stem cells (CSCs). However, little is known about key signals regulating the formation and expansion of CSCs. Moreover, lack of a reliable predictive preclinical model has been a major obstacle to discover new cancer drugs and predict their clinical activity. Here, in ovarian cancer, a highly chemoresistant tumor that is rapidly fatal, we provide the first evidence demonstrating the causal involvement of mechanical stimulus in the CSC phenotype using a customizable microfluidic platform and three-dimensional spheroids, which most closely mimic tumor behavior. We found that ovarian cancer cells significantly acquired the expression of epithelial-to-mesenchymal transition and CSC markers and a remarkable chemoresistance to clinically relevant doses of frontline chemotherapeutic drugs cisplatin and paclitaxel when grown under fluid shear stress, which corroborates with the physiological attainable levels in the malignant ascites, but not under static condition. Furthermore, we uncovered a new link of microRNA-199a-3p, phosphatidylinositol 3-kinase/Akt, and multidrug transporter activation in shear stress-induced CSC enrichment. Our findings shed new light on the significance of hydrodynamics in cancer progression, emphasizing the need of a flow-informed framework in the development of therapeutics.

Ovarian cancer has the highest mortality of all gynecological cancers worldwide^1^. Most of the ovarian cancer patients are diagnosed at an advanced stage (stage III or IV) characterized by large volume of ascites formation and widespread peritoneal dissemination. Despite initial response, over 85% of advanced ovarian cancer patients experience relapse and succumb to the disease. This high degree of inherent and acquired chemoresistance makes treating ovarian cancer particularly challenging (5-year survival rate <25%)^2^. Unravelling the molecular mechanisms contributing to this resistance is critically important.

The discovery of cancer stem cells (CSCs) has advanced our understanding of tumorigenesis and chemoresistance and may provide novel targets for cancer therapy. CSCs have been discovered in various cancers, including ovarian cancer; they have been shown through identifying Hoechst 33342 side population cells or staining for stem cell-specific markers, as well as through enrichment of suspension cultures as spheroids that most closely mimic the advanced stage of ovarian carcinoma present in malignant ascites^3^,^4^,^5^,^6^,^7^. Unlike bulk tumor cells, CSCs are highly tumorigenic and drug resistant, and possess self-renewal capacity. Thus, it is conceivable that tumor recurrence is probably due to the fact that standard chemotherapeutic regimens fail to kill CSCs which can regenerate the entire tumor^8^. However, the key signals regulating the formation and expansion of CSCs remain elusive. This is due mainly to technical limitations in studying CSCs in a sensitive, representative manner in a clinically relevant setting.

Emerging evidence shows that mechanical stimuli, including pressure, stretching, and shear stress, can modulate the morphology, expression of biomarkers, and aggressiveness of tumor cells^9^,^10^,^11^. However, due to its relative difficulty to manipulate and investigate, a dynamic system with fluid flow has been largely neglected thus far, and therefore, CSC phenotypes under flow conditions remain poorly understood. This also highlights some deficiencies in the currently used cell-based screening platforms for new drug discovery and subsequent efficacy testing. Various *in vitro* culture systems have been used to mimic the fluidic flow, including parallel plate device^12^,^13^, rocker plates^12^, spinner flasks^14^, and rotating wall bioreactors^15^. However, those systems have different limitations, such as incapability of generating shear stress as low as physiological values, uneven flow distribution, and difficulty in achieving precise control over the shear stress^16^. Microfluidics is a rising tool used to study the effect of fluid flow on the alteration of cellular behavior. With defined geometry and controlled perfusion flow rate, microfluidic chips provide an *in vitro* cell culture platform that allows precise mimicking of the shear stress in the physiological environment with low cost and reduced sample consumption. However, direct evidence for a role of the CSC phenotype is still lacking. It is also not known whether the enhanced chemoresistance is a consequence of the expansion of the CSC population. Nor is it clear about the underlying mechanisms.

In this study, as a first step to circumvent the above experimental conundrums and to establish a robust framework for analysis of key molecules involved in chemoresistance, we have used a new microfluidic model of the peritoneum, with three-dimensional (3D) spheroids for mimicking tumor behavior. We report for the first time a role of fluid shear stress in CSC phenotypes and chemoresistance which lead to failure of cancer treatment. We also provide a novel link between shear stress, microRNA (miRNA)-199a-3p, and phosphatidylinositol 3-kinase (PI3K)/Akt activation, and ATP-binding cassette G2 (ABCG2) and P-glycoprotein (P-gp) expression in this process. All these suggest a need of the incorporation of physiological flow of body fluids in the screening of cancer drugs. The microfluidic platform may also provide a system for high-throughput analysis more suited as a drug development tool.

## Results

### A new microfluidic model of the peritoneum

**Figure 1:**
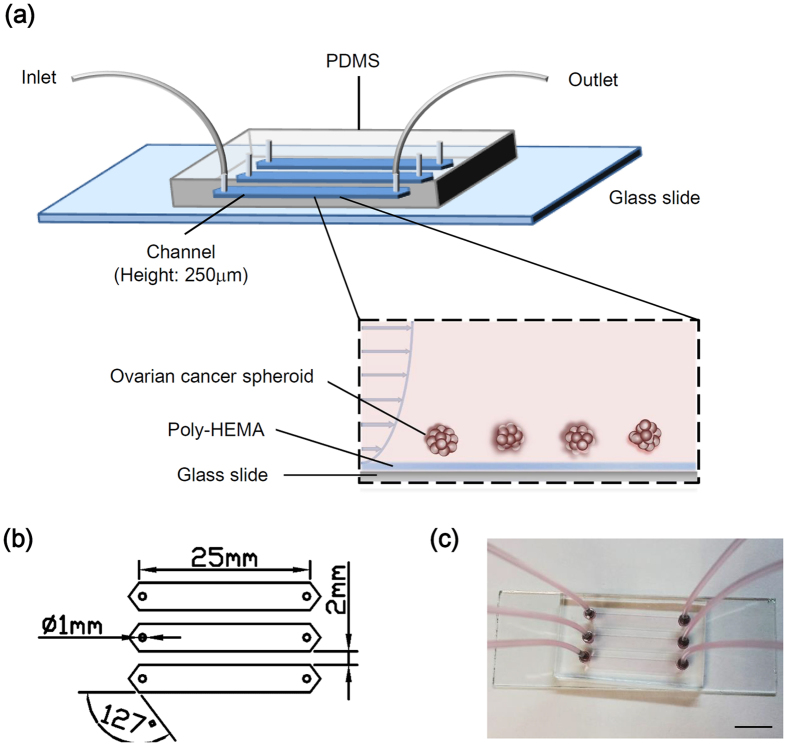
(**a**) Schematic diagram showing the experimental setup and the side view of a poly-HEMA-coated (non-adherent) microfluidic channel under perfusion. **(b)** The dimensions of channels in microfluidic chip. **(c)** Photograph showing the microfluidic chip and experimental setup. Bar, 1 cm.

**Figure 2:**
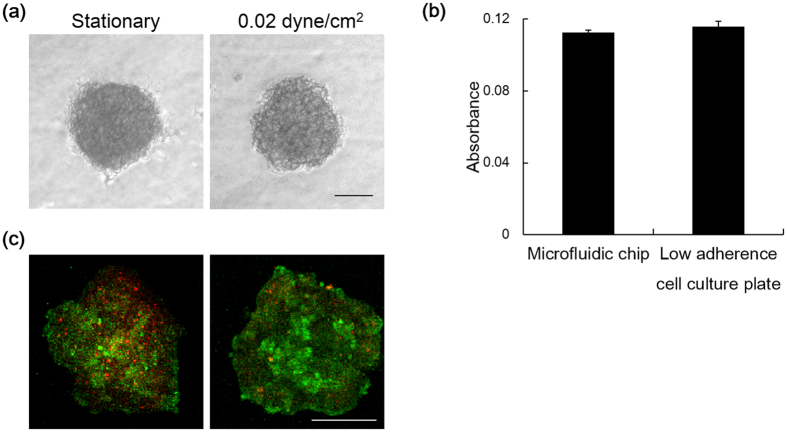
(**a**) Bright field image of ovarian cancer spheroids cultured in microfluidic chip for 24 h under static condition or perfusion with shear stress at 0.02 dyne/cm^2^. Bar, 50 μm. **(b)** Cell viability of spheroids culture under static condition in microfluidic chip and non-adherence cell plate for standard 3D cell culture was determined with MTT assay. The absorbance is presented as mean ± SEM. (**c**) Live/dead fluorescent staining of ovarian cancer spheroids under static condition or perfusion with shear stress at 0.02 dyne/cm^2^. Bar, 50 μm.

Both the dynamic mechanical forces and the 3D environment are very important factors of the tumor progressive cascade. We have developed a customizable microfluidic platform to overcome limitations of conventional strategies and advance studies of the intraperitoneal compartment in tumor aggressiveness. This microfluidic device is advantageous in providing continuous well-defined flow rate to emulate physiological conditions in the peritoneum ascites (Fig. 1). The 3D cell culture is relevant to the clinical situation that the spheroid population (30–200 μm) in the malignant ascites of ovarian cancer patients represents the main source of intraperitoneal outgrowth, and recapitulates the human cancer feature. Moreover, multicellular tumor spheroids are more resistant to drug-induced apoptosis than in monolayers as shown by us and others^17^,^18^,^19^. Due to its nonionic nature, poly-HEMA prevents unphysiological cell attachment to the substratum and matrix deposition, thus keeping tumor spheroids in suspension similar to those isolated from patients’ ascitic fluid (Fig. 1a) It is also worth noting that ovarian cancer spheroids cultured in our microfluidic chip coated with poly-HEMA remained intact without dissociation or adhesion after 24 h of incubation with or without perfusion (Fig. 2a) and their viabilities were similar to spheroids cultured in low adherence plate for standard 3D cell culture (Fig. 2b). However, tumor spheroids were significantly more viable (~50%) with than without perfusion (Fig. 2c).

### Shear stress enhances stemness of ovarian cancer spheroids

**Figure 3:**
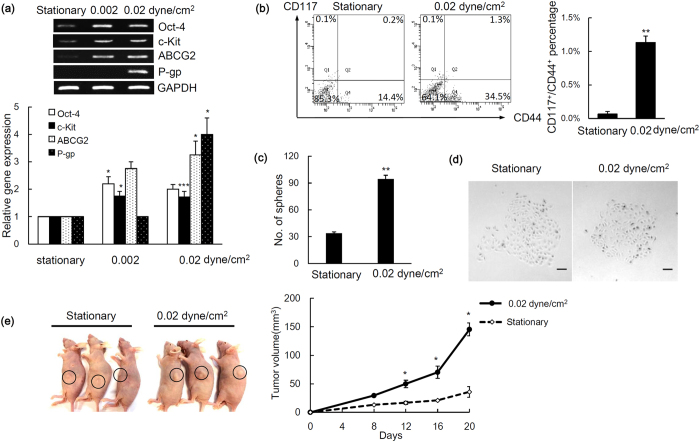
The expression of **(a)** stem cell markers in ovarian cancer spheroid cultured under stationary or perfusion with shear stress at 0.002 or 0.02 dyne/cm^2^ was detected by RT-PCR. The band intensity was determined by densitometry, and data are shown as mean ± SEM. **(b)** Identification of CD117^+^/CD44^+^ cells by FACS. Scatter plots show the coexpression of CD117^+^ and CD44^+^ in spheroids growth under stationary or 0.02 dyne/cm^2^ (left). Percentage of CD117^+^/CD44^+^ cells was showed as mean ± SEM (right). **(c)** The number of secondary tumor spheres formed was counted. Results were presented as the mean ± SEM. **(d)** Spheroids adhere and form colonies under differentiation condition. Bar, 100 μm. **(e)** Xenograft tumor formed after s.c. injection of cells derived from spheroids cultured in microfluidic chips with or without perfusion (left). Tumor growth was measured and represented as mean ± SEM (right). Significant differences between stationary and perfusion culture conditions are indicated with asterisk (*p < 0.05; **p < 0.01; ***p < 0.001).

**Figure 4:**
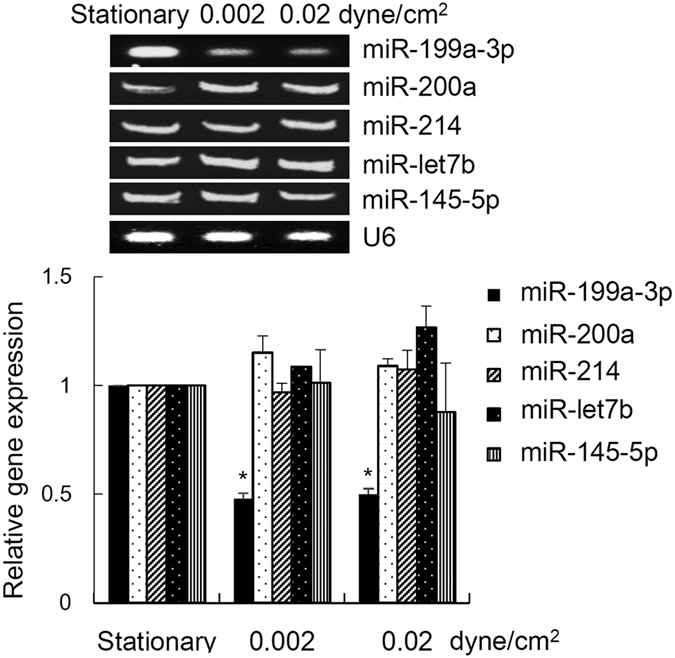
Total RNA was extracted and RT-PCR was performed using sequence-specific primers to miR-199a-3p, miR-200a, miR-214, miR-let7b, and miR-145-5p. U6 was used as an internal control. The band intensity was determined by densitometry and results are presented as mean ± SEM. Significant differences between stationary and shear stress culture are indicated with asterisk (*p < 0.05).

In defining the effects of shear stress on these ovarian cancer spheroids with a focus on stem cell phenotypes, we analysed the expression of stem cell markers. The spheroids were perfused under low shear stress, less than 0.1 dyne/cm^2^, which closely mimics the level expressed in the peritoneum^20^. Moreover, as malignant ascites develop and accumulate during tumor progression, the fluid flow shear stress decreases^20^. We found that spheroids grown under both 0.002 dyne/cm^2^ and 0.02 dyne/cm^2^ induced expression of epithelial-to-mesenchymal markers (Supplementary Fig. S1) and each stem cell marker Oct-4, c-Kit (CD117), ABCG2, and P-gp but not under static conditions (Fig. 3a). Oct-4 is a stem cell marker expressing in CSCs of various cancers. Oct-4 plays an important role during the tumor progression by regulating the tumorigenesis, dedifferentiation, and metastasis^21^,^22^,^23^. Our finding that the expression of Oct-4 was higher at 0.002 dyne/cm^2^ when compared with 0.02 dyne/cm^2^ was consistent with the observed increase in expression of stem cell markers of advanced stage of cancer^24^. c-Kit, receptor of stem cell factor, is identified as an ovarian CSC marker that is highly expressed in recurrent ovarian carcinoma and correlates with poor patient prognosis^25^,^26^,^27^. Our previous study showed that c-Kit regulates ovarian CSC self-renewal and tumorigenesis. Moreover, c-Kit enhances ovarian CSCs resistance to chemotherapeutic drugs through ABCG2, an ABC drug transporter which can rapidly efflux drugs^18^. P-gp is another important marker of drug resistance in ovarian CSCs^28^. Furthermore, we also detected an enrichment of CD117^+^/CD44^+^ population, which possess characteristics of the side population^5^, in spheroids grown under shear stress (Fig. 3b). These spheroids also displayed a greatly enhanced self-renewal potential (Fig. 3c), the ability to differentiate (Fig. 3d), and an increased tumor-initiating capability (Fig. 3e), suggesting that the fluid shear stress facilitates the transition to a stem cell-like status. In search of mechanisms underlying shear stress regulation of the CSC phenotype, we looked into miRNAs, small 21- to 23- nucleotide non-coding RNAs that post-transcriptionally inhibit gene expression by either translation blockage or mRNA degradation. Although many studies have described the roles of miRNAs in the modulation of CSCs^29^, the major physiological and pathophysiological stimuli that induce or suppress CSC gene expression remain incompletely understood. To test this we examined a set of miRNAs, including let-7 family (miR-let7b and miR-145–5p), miR-199a-3p, miR-200a, and miR-214, which have been previously implicated in the CSC phenotype, as well as in the progression and metastasis of ovarian cancer^30^,^31^. Among these miRNAs, only miR-199a-3p was a mechanosensitive miRNA that showed a marked decrease under physiologic shear stress (Fig. 4). Although it is possible that shear stress may affect global miRNA expression, our existing data suggest that not all miRNAs are targets of shear stress. Likewise, miR-199a-3p has been identified as a critical mechanosensitive miRNA, suggesting regulation specificity of mechanical stimuli^32^,^33^.

### Shear stress enhances chemoresistance of cancer spheroids

**Figure 5:**
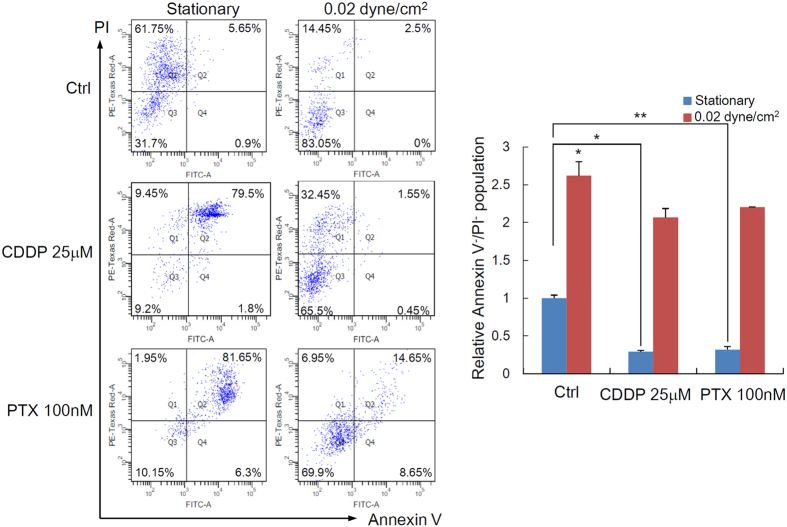
The chemosensitivity of ovarian cancer spheroid under static or 0.02 dyne/cm^2^ shear stress against cisplatin (CDDP) and paclitaxel (PTX) was analysed by Annexin V/PI staining (left). The relative viable cell population (Annexin V^−^/PI^−^ cells) are presented as mean ± SEM (right). Significant differences between untreated and treated spheroids are indicated with asterisk (*p < 0.05; **p < 0.01).

**Figure 6:**
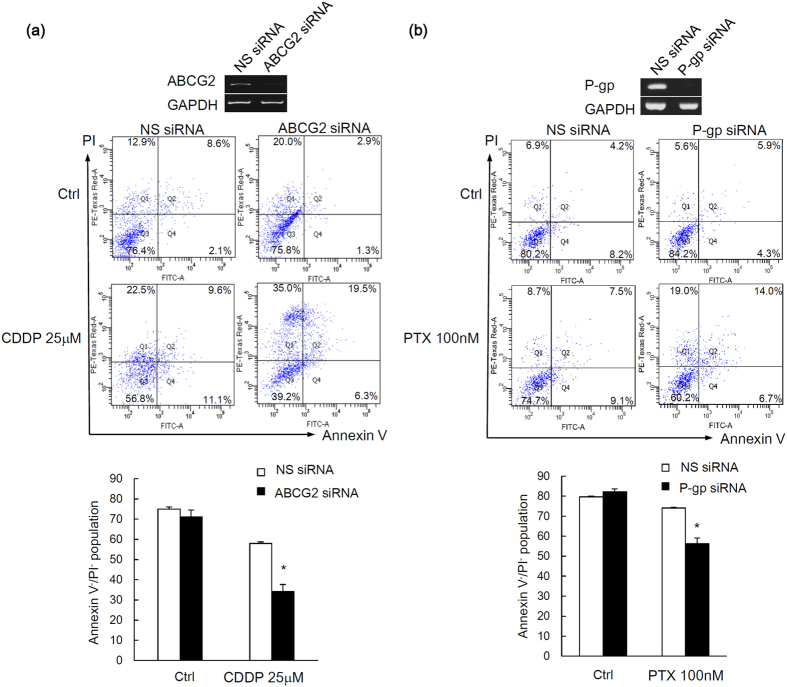
Knockdown of ABCG2 or P-gp enhanced cisplatin/paclitaxel chemosensitivity under shear stress. **(a)** Spheroids transfected with nonspecific (NS), ABCG2, or **(b)** P-gp siRNA and subjected to shear stress against cisplatin (CDDP) or paclitaxel (PTX) were analyzed by Annexin V/PI staining. The relative viable cell population (Annexin V^−^/PI^−^ cells) are presented as mean ± SEM. Significant differences between untreated and treated spheroids are indicated with asterisk (*p < 0.05).

Since the application of wall shear stress increases the expression of ABCG2 and P-gp, we hypothesized that ascitic flow can enhance the chemoresistance of ovarian cancer spheroids. Cisplatin and paclitaxel are the mainstay of the two first-line chemotherapeutic agents currently used for treating unresectable ovarian cancer. To test the hypothesis, ovarian cancer spheroids were treated with doses of cisplatin (25 μM) or paclitaxel (100 nM) relevant to the clinical setting in the presence or absence of wall shear stress. Viable cells are defined as Annexin V^−^/PI^−^ (lower left quadrant). Early apoptotic cells are defined as Annexin V^+^/PI^−^ (lower right quadrant); necrotic cells are defined as Annexin V^−^/PI^+^ (upper left quadrant); late apoptotic cells are defined as Annexin V^+^/PI^+^ (upper right quadrant). Under static condition, cells in tumor spheroids rapidly underwent apoptosis upon cisplatin and paclitaxel treatment with the percentage of viable cells (Annexin V^−^/PI^−^) in spheroids as low as 9.2% (for CDDP) and 10.15% (for PTX), respectively, as shown in Fig. 5. In contrast, under wall shear stress, cells in tumor spheroids showed significantly greater chemoresistance even in the presence of cisplatin and paclitaxel treatment, with 65.59% (for CDDP) and 69.9% (for PTX) of cells in spheroids remained viable, respectively (Fig. 5). Knockdown of ABCG2 or P-gp by small interfering RNA (siRNA) enhanced a cisplatin/paclitaxel-induced cell death under perfusion (Fig. 6), supporting a role for ABCG2 and P-gp in mediating the shear-induced chemoresistance.

### Shear stress regulates stem cell phenotypes and chemoresistance through PI3K/Akt signalling

**Figure 7:**
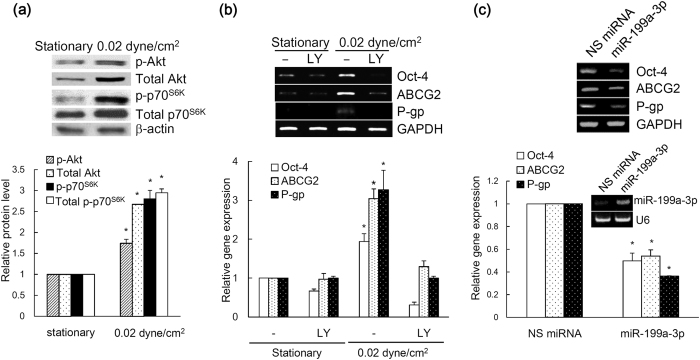
(**a**) The expression of phospho (p)-Akt, Akt, p-p70^S6K^, and p70^S6K^ in ovarian cancer spheroids cultured under static or 0.02 dyne/cm^2^ shear stress were analysed with western blot. The band intensity was determined by densitometry, and data are shown as mean ± SEM. **(b)** Ovarian cancer spheroids were treated with or without 25 μM of LY294002 **(c)** or overexpressed with nonspecific (NS) miRNA or miR-199a-3p were subjected to 0.02 dyne/cm^2^ shear stress. The expression of Oct-4, ABCG2 and P-gp were analyzed by RT-PCR. The band intensity was determined by densitometry, and data are shown as mean ± SEM. Significant differences (*p* < 0.05) between stationary and 0.02 dyne/cm^2^ shear stress are indicated with asterisk.

The molecules and mechanisms underlying mechanotransduction are still largely unknown. Activation of PI3K/Akt signaling is a key step controlling the proliferation and survival of ovarian cancer. In addition, PI3K/Akt signaling is of particular relevance to the chemoresistance^34^, CSC self-renewal^35^, and tumor immune escape^36^. Here, we found that the expression of active (phospho)-Akt in spheroids significantly increased under wall shear stress (Fig. 7a). The shear stress also affected its downstream signaling protein p70^S6K^ (Fig. 7a). To test if PI3K/Akt signaling regulates wall shear stress-induced stemness enrichment, a PI3K/Akt inhibitor LY294002 was used. The addition of LY294002 completely abolished the shear stress-induced expression of Oct-4, ABCG2, and P-gp, whereas there was no significant change observed under static condition (Fig. 7b). Similarly, by overexpressing miR-199a-3p, Oct-4, ABCG2, and P-gp mRNA were downregulated under shear stress (Fig. 7c). Thus, this shear stress-dependent downregulation of miR-199a-3p expression may activate PI3K/Akt signaling^37^,^38^, which subsequently activates the downstream gene expression and stimulates CSC function^30^. While shear stress can activate the MEK/ERK signaling in some other cell models, we ruled out that this possibility since treatment with a specific MEK1 inhibitor (PD98059) in addition to wall shear stress had no effect on blocking the expression of Oct-4, ABCG2 or P-gp (Supplementary Fig. S2). These results suggest that PI3K/Akt is the key signaling pathway mediating the development of ovarian CSC properties and chemoresistance induced by shear stress.

## Discussion

CSC is envisioned to be the key component leading to the relapse of tumor and chemoresistance. However, the knowledge on the establishment of ovarian CSC and its expansion is still limited. In this study, using a microfluidic platform with shear stress and 3D cultures that closely mimic the physiological microenvironment of ovarian cancer, we show for the first time the casual involvement of mechanical stimulus in the pathogenesis of CSCs. We also provide evidence for a role of the miR-199a-3p-PI3K/Akt-ABCG2/P-gp signaling in this process.

Our approach was different from most previous work, which tumor spheroids in those microfluidic systems were trapped and experienced negligible shear stress which was far lower than the physiological condition. Our model, when compared with other approaches, offers a more realistic and clinically relevant experimental model to identify the factors critically involved in the drug resistance. Indeed, we demonstrate that tumor spheroids are more resistant to drug-induced apoptosis when grown under shear stress than nadir levels. These results suggest the significance of shear stress in promoting chemoresistance. Moreover, these results also propose a mechanism that shear stress-induced multiple drug resistance is mediated through activation of ABCG2 and P-gp. This observation can also be seen in most ovarian cancer patients, in which those who develop resistance to single agents impart resistance to several other agents. Our results may also help to explain why, despite encouraging findings in defining the signaling mechanisms of drug-induced apoptosis and resistance in earlier *in vitro* studies, the understanding has not been successfully translated to effective therapies^39^.

These results revealed that peritoneal ascitic flow does not only contribute to the dissemination of ovarian cancer but may also facilitate the enrichment of CSCs. The role of shear stress in maintaining the expansion and the pluripotency has also been observed in pluripotent stem cells^40^,^41^,^42^,^43^, which further support the role of shear stress in the enrichment of the CSC population. These findings are not only relevant to ovarian cancer, but also to other cancers, such as gastric, mesothelioma, and colon cancer, of which peritoneal metastasis is an important pathological process and low shear stress levels characteristic of ascitic flow. In addition, our study provides further understanding of a previously recognized role of shear stress in the regulation of CSC phenotype through the action of miRNA. The fact that one miRNA can target multiple genes suggests a particularly important and broad role for shear stress in the regulation of mRNA levels, which may underlie the known association of the development of ascites and tumor aggressiveness.

In summary, these results provide important insights for therapeutic intervention and reinforce the need to address shear stress effects to improve the current challenge of the low translation rate of anti-cancer therapy from *in vitro* to clinical trials. A cell-line based reliable predictive preclinical model is particularly beneficial for ovarian cancer, which is a rather rare malignancy accounting for ~3% of gynecologic cancers, animal models were unsuitable and there is a limitation for large clinical trials. The microfluidic platform further bolsters the validity of our findings in a high-throughput approach for drug discovery and development.

## Methods

### Cell culture and spheroid formation

Human ovarian carcinoma cell line SKOV-3 was a gift from Dr. N. Auersperg (University Of British Columbia, Vancouver, B. C., Canada). Cells were maintained in M199:MCDB105 (1:1) supplemented with 5% fetal bovine serum (Hyclone), and 100 units/ml penicillin/streptomycin at 37 °C under 5% CO_2_. For spheroid formation, cells were detached from culture dish by trypsinization and plated in 96-well plate coated with 0.5% agarose to prevent cell adhesion. Spheroids were collected for experiments after 48 h. We chose to use the liquid overlay method to ensure the spheroids formed were in uniform shape and size (104.6 ± 1.67 μm), which can effectively maintain the consistency of the physiological gradients of spheroids in each channel. To allow differentiation, spheroids with or without perfusion were plated in adhesive tissue culture dishes under standard culture conditions and cell morphologies were examined 48 h after plating. To analyse for self-renewal capabilities, spheroids with or without perfusion were collected and trypsinized into single cells. Cells were seeded in serum-free medium at a concentration of 5,000 cells/ml and the number of secondary spheres was counted after 72 h.

### Design and fabrication of the microfluidic chip

The microfluidic chip was modified based on previous model published by Rizvi *et al.*^11^. Each microfluidic chip contains three 4 mm (width) × 25 mm (length) × 250 μm (height) channels with 2 mm space in between. A 127° angle was constructed at both ends of the channels to facilitate the entrance and exit of fluid to and from the channel. The inlet and outlet were 1 mm in diameter and were 5 mm from the edge of the channel (Fig. 1b). The microfluidic chip was fabricated using polydimethylsiloxane (PDMS; Dow Corning) by standard soft-lithography technique. A silicon wafer (University Wafer) was spin-coated with a layer of SU-8 2075 photoresist (Microchem, Corp.) of height 250 μm and was soft-baked before UV exposure. The device pattern was prepared with AutoCAD 2012 (Autodesk Inc.) and printed on a photofilm for UV exposure on the photoresist, followed by post-exposure-bake and development to obtain a master mold. The PDMS and curing agent (Sylgard 184) mix (10:1, w/w) was poured onto the silanized mold to create a depth with around 5 mm. After degassing under vacuum, the PDMS was allowed to polymerize under 65 °C for at least 2 h. The polymerized PDMS with the channel pattern was gently peeled from the mold after cooling. The channels were punched with 1 mm-diameter biopsy punch (Miltex) to create inlet and outlet before bonding to a glass slide by plasma treatment. The bonded device was heated at 150 °C for 1 h to recover the hydrophobicity before use.

### Spheroid loading and experimental set up

The channels in chip were sterilized with 75% ethanol and then flushed with PBS before use. To prevent undesired spheroid adhesion in channels, the channels were filled with 12 mg/ml of poly 2-hydroxyethylmethacrylate (poly-HEMA; Sigma) in 95% ethanol and allowed to dry in tissue culture hood overnight under flow. Prior to spheroid loading, the channels coated with poly-HEMA were flushed with PBS. Spheroids were collected and washed three times with serum-free medium before loading. Sixty spheroids resuspended in serum-free M199:MCDB105 were loaded into each channel with pipette tip. The channels were connected to a multi-syringe pump (Cole Parmer) equipped with syringes and gas-permeable silicon tubings containing serum-free medium, chemotherapeutic drugs, or inhibitor (LY294002, 25 μM; Calbiochem). Spheroids were perfused at flow rate 30.1 μl h^−1^ (0.002 dyne cm^−2^) or 301.2 μl h^−1^ (0.02 dyne cm^−2^) for 24 h at 37 °C and 5% CO_2_. The wall shear stress was calculated with the following equation:


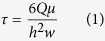


where τ–wall shear stress; *Q*–flow rate; *μ*–viscosity (0.01 g cms^−1^); *h*–height of channel; and *w*–width of channel. For static cell culture, the inlet and outlet of the channels were sealed with tape after spheroid loading so that the gas diffusion efficiency in static group would be the same as the perfusion group.

### Simulation of flow field and shear stress

**Figure 8:**
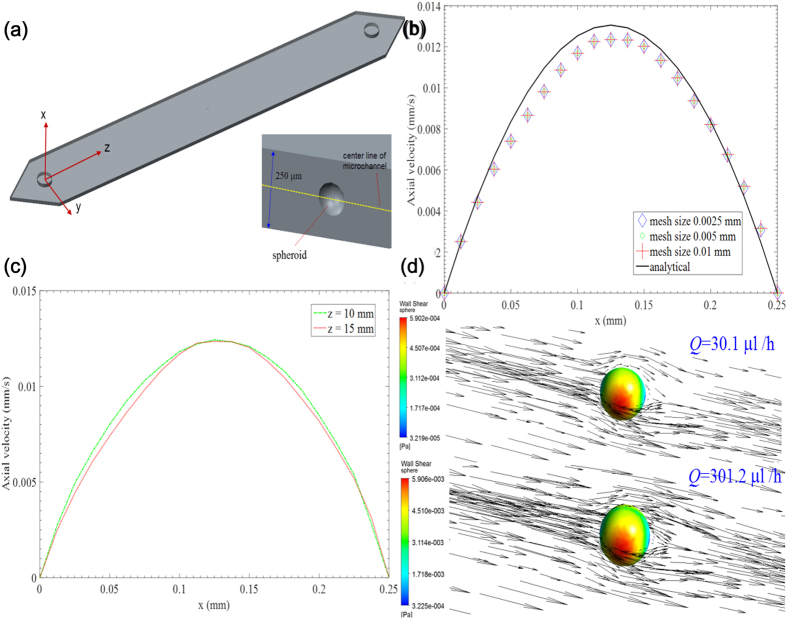
(**a**) 3D model and the coordinate system of the microchannel, with inset showing the location of spheroid with respect to the channel. **(b)** The comparison between CFD solution using different mesh sizes and analytical solution of axial velocity profile across channel height. (**c)** The fully developed parabolic profile of axial velocity across channel height at different downstream locations. **(d)** The velocity vectors of flow passing around the spheroid, and the shear stress distribution over spheroid surface, at different flow rates.

Three microchannels were identical in the experiment; thus only one channel was adopted in the numerical model where the case of one spheroid suspended in serum-free M199:MCDB105 medium was considered (Fig. 8). The medium was assumed to possess Newtonian incompressible property. The gravity effect was neglected for the sake of simplicity. The simulations were performed using numerical grids composed of tetrahedron elements generated by ANSYS package ICEM CFD 15.0. The flow field and shear stress problem was governed by Navier–Stokes equation, which was solved by CFD software Ansys Fluent 15.0. The governing equations were discretized to algebraic equations using a control-volume-based technique. SIMPLE (Semi-Implicit Method for Pressure-Linked Equations) scheme was applied for velocity-pressure coupling. The spatial discretization schemes were set as least squares cell based scheme for gradient, second-order scheme for pressure, and second-order upwind scheme for momentum. An iterative solver was deployed to solve the control-volume discretized equations. The iterative time step was 10^−3^ s and the solution converged when the residual was below a tolerance set as 1.0 × 10^−6^. No-slip condition was applied at the solid boundaries of the walls of the microchannel, as well as the surface of the spheroid. Zero gauge pressure was applied at the outlet of the channel. Laminar flow conditions were used, and flow velocity was specified at the inlet of the channel. The numerical data were subsequently analyzed by the Ansys CFX-Post Processor 15.0 after the simulation was completed. Two flow rates, 30.1 and 301.2 μl/h, were used in the simulation. Each case was simulated for three times, and the relative difference was less than 1%. Different mesh sizes were used in simulation. Using the coordinates shown in Fig. 8a, the theoretical velocity solution of the axial flow velocity with a no-slip boundary condition can be described by^44^,


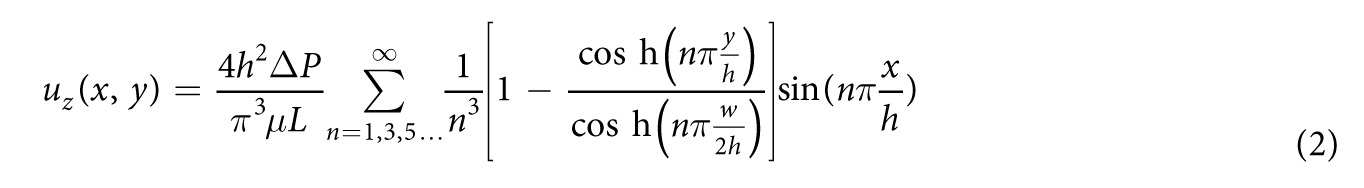


where Δ*P* represents the pressure drop, which can be correlated with flow rate, *Q*, by,


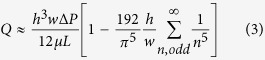


The analytical solution was solved by Matlab, and compared with our simulation results of the axial flow velocity across channel height in the middle of channel where y = 0. A good agreement has been found except for a deviation in maximum velocity, as shown in Fig. 8b. The analytical solution is derived from a straight channel, which is not exactly same as the channel in our case; this may contribute to the deviation. A mesh size of 0.05 mm was used in the following simulation to reduce computational cost. The fluid flow has become fully developed with parabolic profile (Fig. 8c). The averaged shear stresses over the surface of spheroid were 0.00390268 and 0.0390525 dyne/cm^2^, at flow rate of 30.1 and 301.2 μl/h, respectively (Fig. 8d).

### MTT assay

The viability of cells in spheroids cultured in microfluidic chip or in low adherence plate used for standard static 3D culture was assessed by colorimetric MTT (3-(4,5-dimethylthiazol-2-yl)-2,5-diphenyltetrazolium bromide) assay according to the manufacturer’s instructions (Sigma). In brief, after 24 h of incubation, ovarian cancer spheroids were collected and trypsinized into single cells suspension. Same number of cells from spheroid were resuspended in MTT solution and incubated at 37 °C for 2 h. Unadhered cells were collected by centrifugation and cells were dissolved with DMSO. Colorimetric analysis was performed at wavelength 595 nm with reference 625 nm using a microplate reader (Bio-Rad).

### Live/dead fluorescent staining

Ovarian cancer spheroids with or without perfusion were collected and stained with Calcein AM (5 μM; Invitrogen) and propidium iodide for 30 min. The staining was imaged by confocal microscopy (Carl Zeiss) at 10 different focal planes with 10 μm apart. Reconstruct images were stacked by Zen software (Carl Zeiss).

### Polymerase chain reaction (PCR)

The expression level of stem cell markers were analysed by RT-PCR. In brief, total RNA was extracted with Trizol and reverse transcribed using the first-stranded cDNA synthesis kit (Invitrogen). The target genes were amplified with the specific primers as follows: Oct-4: sense 5′-ATCCTGGGGGTTCTATTTGG-3′, antisense 5′-TCTCCAGGTTGCCTCTCACT-3′; c-Kit: sense 5′-TCATGGTCGGATCACAAAGA-3′, antisense 5′-AGGGGCTGCTTCCTAAAGAG-3′; ABCG2: sense 5′-CTGAGATCCTGAGCCTTTGG-3′, antisense 5′-TGCCCATCACAACATCATCT-3′; P-gp: sense 5′-GAGCCTACTTGGTGGCACAT-3′, antisense 5′-TCCTTCCAATGTGTTCGGCA-3′; Snail: sense 5′-TTCCAGCAGCCCAACGACCAG-3′ and antisense 5′-CGGACTCTTGGTGCTTGTGGA-3′; Slug: sense 5′-ACGCCTCCAAAAAGCCAAAC-3′, antisense 5′-GGTAATGTGTGGGTCCGAAT-3; N-cadherin: sense 5′-CACTGCTCAGGACCCAGAT-3′ and antisense 5′-TAAGCCGAGTGATGGTCC-3′; E-cadherin: sense 5′-GGGTGACTACAAAATCAATC-3′ and antisense 5′-GGGGGCAGTAAGGGCTCTTT-3′; and GAPDH: sense 5′-ATGTTCGTCATGGGTGTGAACCA-3′, antisense 5′-TGGCAGGTTTTTCTAGACGGCAG-3′. To analyse the expression of microRNAs, RT-PCR was carried out using Taqman microRNA reverse transcription kit (Applied Biosystems) and the following stem-loop primers: miR-199a-3p, 5′-GTTGGCTCTGGTGCAGGGTCCGAGGTATTCGCACCAGAGCCAACTAACCA-3′; miR-200a, 5′-GTTGGCTCTGGTGCAGGGTCCGAGGTATTCGCACCAGAGCCAACACATCG-3′; miR-214, 5′-GTTGGCTCTGGTGC AGGGTCCGAGGTATTCGCACCAGAGCCAACACTGCC-3′; miR-let7b, 5′-GTTGGCTCTGGTGCAGGGTCCGAGGTATTCGCACTGGATGCGACAATCAC-3′; miR-145-5p, 5′-GTTGGCTCTGGTGCAGGGTCCGAGGTATTCGCACCAGAGCCAACAGGGAT-3′; and U6, 5′-GTCGTATCCAGTGCAGGGTCCGAGGTATTCGCACTGGATACGACAAAAATAT-3′. U6 was used as an internal control.

### Stem cell marker staining

Ovarian cancer spheroids cultured in microfluidic chip were dissociated into single cells and double-labeled with mouse anti-human CD117-PE and mouse anti-human CD44-FITC (BD Biosciences, San Jose, CA) following the manufacturer’s instruction. Percentage of CD117^+^/CD44^+^ cells was analyzed using BD FACSAria^TM^ III (BD Biosciences).

### *In vivo* xenograft experiment

Ovarian spheroids cultured in microfluidic chip were collected and dissociated into cells. 2 × 10^6^ cells were suspended in 50 μl of PBS and injected subcutaneous into the lower flank of 5- to 7-week-old female athymic nude mice (BALB/c-nu/nu). Tumour size was monitored twice weekly. Tumour volume was calculated as tumor volume [mm^3^] = (length [mm]) × (width [mm])^2^ × 0.52. Mice were sacrificed when tumor reached a diameter of 1 cm. All animal studies were conducted according to the protocols approved by the Committee on the Use of Live Animals in Teaching and Research at the University of Hong Kong.

### Chemosensitivity analysis

The chemosensitivity against cisplatin (CDDP, 25 μM; Calbiochem) and paclitaxel (PTX, 100 nM; Calbiochem) was analysed by AnnexinV/propidium iodide (PI) staining (Annexin-V-FLUOS Staining kit; Roche). After 24 h of incubation with or without perfusion, spheroids in channels were collected and trypsinized into single cells suspension. The cells were stained following the manufacturer’s instruction and the fluorescent intensity was analysed by BD FACSAria^TM^ III (BD Biosciences). The Annexin V^−^/PI^−^ cells were determined as viable cells.

### siRNA and miRNA transfection

Transfection of the ABCG2 (5′-GCAGAUGCCUUCUUCGUUA-3′) and P-gp siRNA (5′-GAGCUUAACACCCGACUUA-3′) (20 nM), (Dharmacon, Lafayette, CO, USA) was conducted using SilentFect (Bio-Rad) according to the manufacturer’s instructions. Non-targeting siRNA duplex (Dharmacon, Lafayette, CO, USA) was used as control. MiR-199a-3p mimic or nonspecific miRNA (100 nM), (RiboBio, China) were transfected with SilentFect according to the manufacturer’s protocol.

### Western blot analysis

Shear stress-induced molecular signaling activation was analysed by western blotting. After perfusion, spheroids were collected from channels and lysed with 1X sodium dodecyl sulfate (SDS) sample buffer. Equal amount of protein were resolved by SDS-PAGE and transferred to nitrocellulose membrane. Membranes were blocked with 5% non-fat milk, and then incubated with anti-phospho-Akt (1:1,000), anti-Akt (1:1,000), anti-phospho-p70^S6K^ (1:1,000), and anti-p70^S6K^ (1:1,000) (Cell Signaling) overnight at 4 °C. β-actin (1:5,000; Sigma) was used as a loading control. The protein-antibody complexes were detected by horseradish peroxidase-conjugated secondary antibodies followed by the enhanced chemiluminescence (Amersham).

### Statistical analysis

*P* values were based upon Student’s t test using GraphPad Prism. *P* < 0.05 was considered to be statistically significant on the basis of at least three independent sets of experiments.

## Additional Information

**How to cite this article**: Ip, C. K. M. *et al.* Stemness and chemoresistance in epithelial ovarian carcinoma cells under shear stress. *Sci. Rep.* **6**, 26788; doi: 10.1038/srep26788 (2016).

## Supplementary Material

Supplementary Information
