# Supplementary material for: Stemness and chemoresistance in epithelial ovarian carcinoma cells under shear stress
Source: Sci Rep. 2016 Jun 1;6:26788. doi: 10.1038/srep26788 (PMC4887794; doi:10.1038/srep26788)
Supplement: Supplementary Information [file srep26788-s1.doc]

**SUPPLEMENTARY INFORMATION**

**Stemness and chemoresistance in epithelial ovarian carcinoma cells under shear stress**

**Carman K. M. Ip^1,†^, Shan-Shan Li^1,†^, Matthew Y. H. Tang^2^, Samuel K. H. Sy^2^, Yong Ren^3^, Ho Cheung Shum^2,*^, and Alice S. T. Wong^1,*^**

^1^School of Biological Sciences, University of Hong Kong, Pokfulam Road, Hong Kong.

^2^Department of Mechanical Engineering, University of Hong Kong, Pokfulam Road, Hong Kong.

^3^Department of Mechanical, Materials & Manufacturing Engineering, University of Nottingham Ningbo China, China.

^†^These authors contributed equally to this work.

^*^Correspondence: Alice S. T. Wong (Email: awong1@hku.hk); Ho Cheung Shum (Email: ashum@hku.hk).

This file includes supplementary figures S1-S2

**
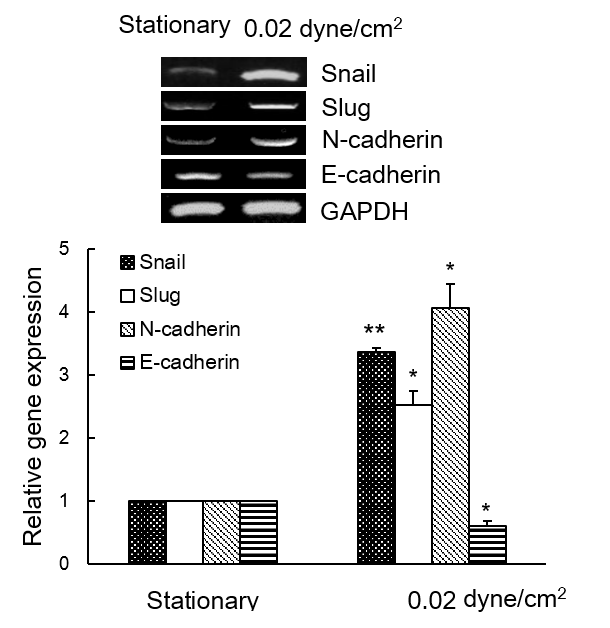
**

**Figure S1:** Ovarian cancer spheroids were cultured under stationary or perfusion with shear stress at 0.02 dyne/cm^2^. The expression of Snail, Slug, E-cadherin, and N-cadherin were analyzed by RT-PCR. The band intensities were determined by densitometry and results are shown as mean±SEM. Significant differences between stationary and shear stress culture are indicated with asterisk (*, *p*<0.05; **, *p*<0.01 ).

**
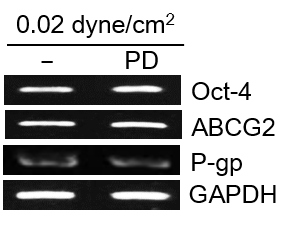
**

**Figure S2:** Ovarian cancer spheroids were treated with or without 50 μM of PD98059 in addition to 0.02 dyne/cm^2^ shear stress. The expression of Oct-4, ABCG2 and P-gp were analyzed by RT-PCR.
